# Soluble Thrombomodulin as a Marker of Endothelial Injury in Early Post-Transplant Period: A Comparative Study of Simple Hypothermia and Pulsatile Machine Perfusion in Kidney Graft Preservation

**DOI:** 10.3390/jcm15010269

**Published:** 2025-12-29

**Authors:** Maciej Kotowski, Anna Prekwa, Adam Nowacki, Iga Stukan, Karol Tejchman, Jerzy Sieńko, Przemysław Nowacki, Bogusław Machaliński, Marek Ostrowski

**Affiliations:** 1 Department of General Surgery and Transplantation, Pomeranian Medical University, 70-111 Szczecin, Poland; maciej.j.kotowski@gmail.com (M.K.); annaprekwa@gmail.com (A.P.); ktejchman78@gmail.com (K.T.); mostrowski@poczta.onet.pl (M.O.); 2 Department of Vascular Surgery, General Surgery and Angiology, Pomeranian Medical University, 70-111 Szczecin, Poland; itskorn@gmail.com; 3 Department of General Pathology, Pomeranian Medical University, 70-111 Szczecin, Poland; boguslaw.machalinski@pum.edu.pl; 4 Department of General, Transplant and Liver Surgery, Pomeranian Medical University, 71-455 Szczecin, Poland; jsien@poczta.onet.pl; 5 Department of Neurology, Pomeranian Medical University, 71-252 Szczecin, Poland; nowackiprz@gmail.com

**Keywords:** kidney transplantation, machine perfusion, reperfusion injury, thrombomodulin, endothelial injury, cold ischemia, graft function, biomarkers

## Abstract

**Background**: Ischemia–reperfusion injury is a major contributor to early graft dysfunction after kidney transplantation and is associated with endothelial damage, reflected by circulating soluble thrombomodulin (sTM). This exploratory study aimed to assess very early graft-level changes in renal vein sTM during reperfusion using a paired-kidney design, in which kidneys from the same donor were preserved using different strategies: static cold storage (SCS) and hypothermic machine perfusion (HMP). **Methods**: Renal vein blood samples were collected intraoperatively at 1 and 30 min after reperfusion. Plasma sTM concentrations were determined using ELISA. Early graft function was monitored during the first 7 days post-transplantation. **Results**: Cold ischemia time was longer in the HMP group than in the SCS group (20 ± 8 h vs. 13 ± 6 h, *p* < 0.05). At 1 min post-reperfusion, sTM levels were comparable between groups. In the HMP group, sTM decreased significantly between 1 and 30 min after reperfusion, whereas no change was observed in the SCS group. Between-group differences at either time point did not reach statistical significance. Early renal function parameters improved in both groups, with no significant inter-group differences. No cases of delayed graft function or graft thrombosis occurred. **Conclusions**: Kidney preservation strategy may modulate very early graft-level endothelial responses during reperfusion, reflected by renal vein sTM dynamics. Although a limited sample size may have reduced the ability to detect between-group differences, very early renal vein sTM measurements may provide insight into ischemia–reperfusion injury. Clinical relevance requires validation in larger studies.

## 1. Introduction

Chronic Kidney Disease (CKD) is characterized by a progressive decline in renal function, driven by a cascade of pathological changes [1]. In Poland, an estimated four million people are affected by this condition, many of whom remain unaware until the disease reaches advanced stages [2]. For patients with end-stage renal disease (ESRD), kidney transplantation remains the preferred renal replacement therapy, offering superior survival and quality of life compared with long-term dialysis [3].

Kidney transplantation represents a critical therapeutic option for ESRD, yet its success hinges on minimizing ischemia–reperfusion injury (IRI) during organ procurement and preservation [4,5]. Warm and cold ischemic periods, inherent to organ retrieval and storage, compromise graft viability, particularly through endothelial damage [6,7]. Two preservation strategies predominate: static cold storage (SCS), a cost-effective method limited to 24 h [8], and hypothermic machine perfusion (HMP), a dynamic approach that extends preservation to 48 h by mimicking physiological conditions [9,10]. While both methods facilitate organ allocation and recipient preparation, their comparative impact on endothelial integrity and graft outcomes remains underexplored.

**Figure 1:**
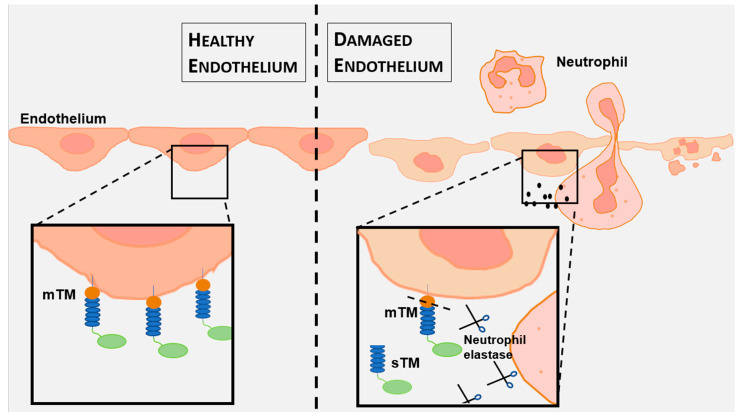
Cleavage of membrane thrombomodulin by neutrophil elastase in endothelial damage. Schematic representation of thrombomodulin dynamics in healthy (**left**) and damaged (**right**) endothelium. In healthy endothelium, membrane-bound thrombomodulin (mTM) is anchored in the plasma membrane, promoting anticoagulation via thrombin binding and protein C activation. In damaged endothelium, neutrophil elastase cleaves mTM at the serine/threonine-rich domain, releasing soluble thrombomodulin (sTM) into the extracellular space, a hallmark of endothelial injury.

Soluble thrombomodulin (sTM), a fragment of the transmembrane glycoprotein thrombomodulin, emerges as a critical indicator of endothelial injury in kidney transplantation [11,12,13]. Released into the plasma following proteolytic cleavage triggered by endothelial damage, sTM reflects the oxidative and inflammatory stresses that threaten graft viability (Figure 1) [14,15]. Its elevation in the early post-transplant period signals IRI, positioning sTM as a promising biomarker to guide preservation strategies and optimize transplant outcomes. However, the relationship between preservation techniques, sTM levels, and renal function has not yet been fully elucidated.

Events occurring immediately after reperfusion are increasingly recognized as critical determinants of endothelial injury and microvascular dysfunction in transplanted organs. Experimental and clinical studies suggest that biomarkers measured within the initial minutes of reperfusion may capture preservation-related effects that are not detectable at later time points using conventional functional endpoints [16,17]. Therefore, the aim of this study was to evaluate early changes in renal vein sTM during the first 30 min of reperfusion in kidney transplant recipients and to compare endothelial injury between kidneys preserved by SCS and HMP. Early renal function was evaluated during the first 7 days post-transplantation, together with exploratory analyses of the associations between sTM, cold ischemia time, and selected clinical parameters. We hypothesized that HMP would be associated with more favorable sTM dynamics, reflecting reduced endothelial injury, despite potential differences in cold ischemia time.

## 2. Materials and Methods

### 2.1. Materials

Blood samples were collected from 40 kidney transplant recipients. Two intraoperative samples (7.5 mL each) were obtained from the renal vein of the transplanted kidney at 1 and 30 min post-reperfusion. Patients were divided into two groups (*n* = 20 each): one group received kidneys preserved by hypothermic machine perfusion (HMP) and the other received kidneys preserved by static cold storage (SCS). Each sample was analyzed for plasma-soluble thrombomodulin (sTM) concentration.

The observation period in the present study was limited to the immediate intraoperative phase and the first 7 days post-transplantation. Long-term graft function and survival data were not systematically collected for this historical cohort and, therefore, were not analyzed.

### 2.2. Kidney Procurement, Preservation, and Transplantation

This study included patients who underwent kidney transplantation at the Department of General and Transplant Surgery, Independent Public Clinical Hospital No. 2 in Szczecin, Poland, between 2014 and 2016. Kidneys were procured from brain-dead donors, with only paired kidneys being included. Multi-organ retrievals were excluded due to varying perfusion fluids.

Kidneys were procured using Custodiol HTK (Dr. Franz Köhler Chemie GmBH, Alsbach-Hähnlein, Germany) solution at 4 °C. One kidney from each donor was assigned to SCS and the other to HMP (LifePort^®^, Organ Recovery Systems; Itasca, IL, USA). Allocation was pragmatic and ethically driven based on clinical workflow. Kidneys were transported to the hospital and stored until transplantation. Recipients were selected based on the Poltransplant National Waiting List.

Transplantation was performed via a retroperitoneal approach, with SCS kidneys transplanted first, followed by HMP kidneys. Renal vessels were anastomosed end to side to the external iliac vessels, and the ureter was anastomosed to the bladder. Following reperfusion, two 7.5 mL blood samples were collected from the renal vein at 1 and 30 min, stored in EDTA tubes on ice, and analyzed for sTM.

The study included 17 women and 23 men (recipients). The mean time of cold ischemia was 15.85 h, and the mean warm ischemia time was 24.8 min. Recipients received immunosuppressive therapy.

One kidney from each deceased brain-dead donor was allocated to SCS and the contralateral kidney to HMP in a 1:1 fashion. Allocation was performed according to the center’s routine paired-kidney assignment procedure. Recipients were selected from the national waiting list (Poltransplant) according to standard matching criteria.

Kidneys allocated to SCS were stored in preservation solution on ice until implantation. Kidneys allocated to HMP were connected to a LifePort^®^ hypothermic machine perfusion device (Organ Recovery Systems, Itasca, IL, USA) and perfused with preservation solution at 4 °C in a pulsatile, pressure-controlled mode using settings recommended by the manufacturer. Perfusion pressure and flow were continuously monitored, and machine-derived vascular resistance was recorded.

At our center, kidneys preserved by SCS were transplanted first, followed by those preserved by HMP, reflecting logistical and ethical considerations, including the immediate availability of SCS grafts and the time required to connect and stabilize the HMP circuit. Consequently, cold ischemia time (CIT) was significantly longer in the HMP group than in the SCS group (Figure S1). This difference did not affect sTM levels (Figure S2).

### 2.3. ELISA Assay

Plasma-soluble thrombomodulin concentrations were measured using commercially available enzyme-linked immunosorbent assay (ELISA) according to the manufacturer’s instructions (Quantikine Human Immunoassays, R&D Systems, Minneapolis, MN, USA). Briefly, samples diluted 1:20 were incubated on 96-well plates coated with monoclonal antibodies specific to sTM for 2 h at room temperature. After washing, a secondary monoclonal antibody conjugated with horseradish peroxidase (HRP) was added, followed by TMB substrate. The reaction was stopped with 2N sulfuric acid, and absorbance was measured at 450 nm using a Microplate Reader ELX 808IU (Bio-Tek Instruments Inc., Winooski, VT, USA). sTM concentrations were determined using a quadratic or four-parameter standard curve, following the manufacturer’s instructions. According to the manufacturer, the lower limit of detection for this assay is 27 pg/mL, and the mean intra- and inter-assay coefficients of variation are below 2.9% and 6.9%, respectively.

### 2.4. Statistical Analysis

Statistical analyses were performed using IBM SPSS Statistics (software ver. 25, IBM Corporation, Armonk, NY, USA) and GraphPad Prism version 5.0 (GraphPad Software Inc., San Diego, CA, USA). Due to non-normal data distribution (Shapiro–Wilk test), descriptive statistics (median, mean, minimum, maximum) were used. Between-group comparisons of independent variables were conducted using the Mann–Whitney U test, while paired variables were analyzed using the Wilcoxon signed-rank test. Comparisons of multiple dependent samples were performed using Friedman’s ANOVA and Kendall’s coefficient of concordance. Correlations were assessed using Spearman’s rank correlation coefficient. A significance level of *p* < 0.05 was adopted.

In addition, cold ischemia time (CIT) was compared between groups, and its association with sTM concentrations at 30 min post-reperfusion was explored using Spearman’s rank correlation within each preservation group. Given the limited sample size and single-center, exploratory design, the study was not powered to detect modest between-group differences, and the results should be interpreted as hypothesis-generating.

### 2.5. Ethical Approval and Informed Consent

The study was conducted in accordance with the Declaration of Helsinki and was approved by the Bioethics Committee of the Pomeranian Medical University in Szczecin, Poland (KB-0012/19/13, 21 January 2013). Written informed consent was obtained from all transplant recipients prior to study inclusion. Organ procurement and transplantation procedures were performed in accordance with national legislation and European guidelines; donor families were informed in line with the standard procedures of the national organ donation program.

## 3. Results

Baseline characteristics, including sex, age, body mass index (BMI), duration of hemodialysis, HLA mismatches (A, B, DR), total HLA mismatch count, HLA score, serum creatinine, urea, potassium, estimated glomerular filtration rate (eGFR), white blood cell count (WBC), platelet count, and hematocrit, were compared between groups. No statistically significant differences were observed for any parameter (*p* > 0.05; Table 1), indicating that both groups were comparable in terms of demographic and clinical characteristics prior to transplantation. This homogeneity supports the validity of subsequent comparisons between preservation methods.

In this paired-kidney design, kidneys preserved by SCS and HMP originated from the same donors, resulting in comparable donor characteristics between groups (Table S1). As expected, based on the fixed transplantation order and the additional time required to connect kidneys to the perfusion device, CIT was significantly longer in the HMP group than in the SCS group (20 ± 8 h vs. 13 ± 6 h; *p* < 0.05, Figure S1).

**Figure 2:**
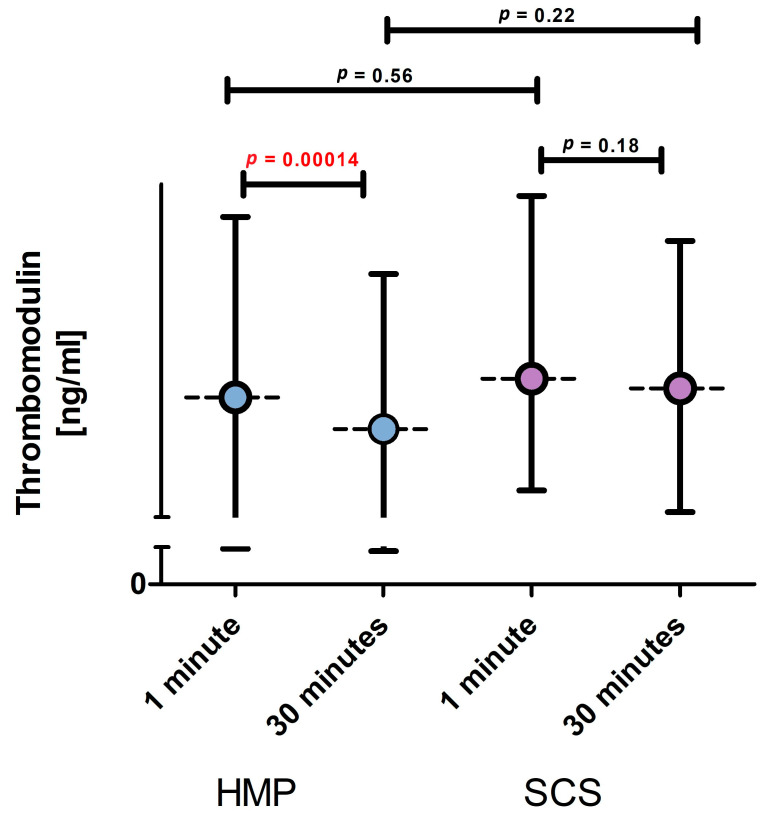
Mean plasma soluble thrombomodulin (sTM) concentrations at 1 min and 30 min post-reperfusion in the HMP group (blue; *n* = 20) and SCS group (pink; *n* = 20). Horizontal dashed lines represent mean values, and vertical bars indicate minimum and maximum concentrations. Data are shown as a mean dashed line, with vertical bars representing minimum and maximum values. Statistical significance (*p*-value) is shown, with significant value (*p* < 0.05) highlighted in red (Wilcoxon matched-pairs test; Statistica software ver. 25).

Plasma sTM concentrations were compared between the SCS (*n* = 20) and HMP (*n* = 20) groups at 1 and 30 min post-reperfusion (Figure 2). At 1 min post-reperfusion, sTM levels did not differ significantly between groups (SCS: mean 27.5 ng/mL, range 17.4–44.0 ng/mL; HMP: mean 25.8 ng/mL, range 14.4–42.1 ng/mL). At 30 min post-reperfusion, sTM levels were lower in the HMP group (mean 23.0 ng/mL, range 13.5–37.0 ng/mL) than in the SCS group (mean 27.5 ng/mL, range 15.5–39.9 ng/mL); however, this difference did not reach statistical significance. Within the HMP group, a significant reduction in sTM concentrations was observed between 1 min and 30 min post-reperfusion (25.8 ng/mL vs. 22.9 ng/mL), whereas no significant change was observed in the SCS group (27.5 ng/mL vs. 26.6 ng/mL). Given the significant difference in CIT between groups (Figure S1), the relationship between CIT and sTM concentrations at 30 min post-reperfusion was explored within each preservation group (Figure S2). In the HMP group, no significant correlation was observed (Spearman’s r = −0.13; *p* = 0.13). In the SCS group, a trend toward a moderate negative correlation between CIT and sTM levels was noted; however, this association was not statistically significant (r = −0.5525; *p* = 0.55). These findings suggest that CIT alone does not fully explain the observed sTM dynamics, particularly in the HMP group.

**Figure 3:**
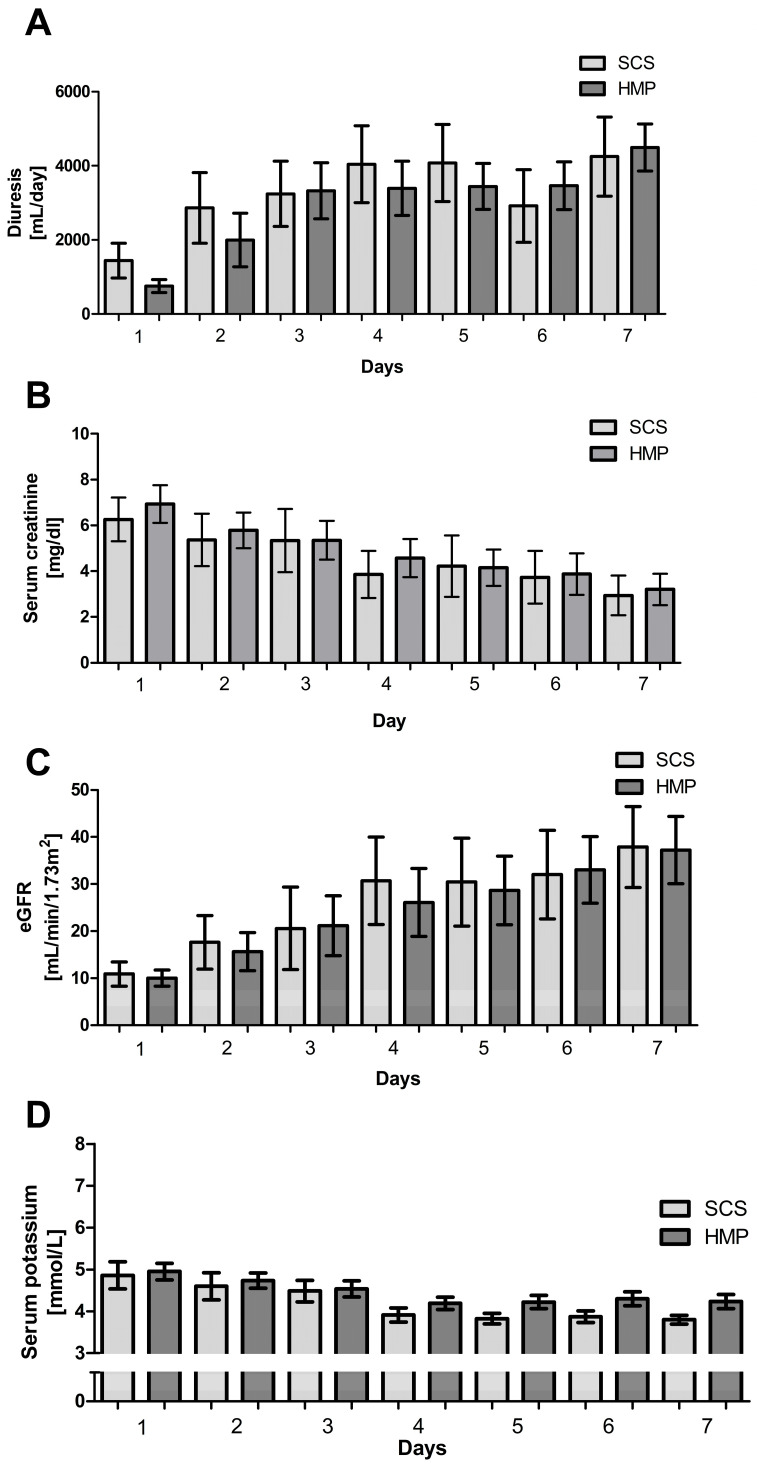
Renal function parameters, including diuresis (**A**), serum creatinine (**B**), estimated glomerular filtration rate (eGFR) (**C**), and serum potassium (**D**), were assessed in the SCS and HMP groups (*n* = 20 each) over the first seven days post-transplantation. Data are shown as mean ± SEM. All parameters exhibited a significant decline across successive days (*p* < 0.01; Friedman ANOVA, Kendall’s coefficient of concordance, Wilcoxon matched-pairs test; Statistica software ver. 25).

Renal function parameters, including diuresis (Figure 3A), serum creatinine (Figure 3B), estimated glomerular filtration rate (eGFR) (Figure 3C), and serum potassium (Figure 3D), were assessed in both groups over the first 7 days post-transplantation. Within each group, all parameters showed significant changes over time (*p* < 0.01; Friedman ANOVA, Kendall’s coefficient of concordance, Wilcoxon matched-pairs test; Figure 3). No cases of delayed graft function, defined as the requirement for dialysis during the first post-transplant week, or graft thrombosis were observed in either group. Between-group comparisons revealed no statistically significant differences in renal function parameters at any point during the observation period (Mann–Whitney U test, *p* > 0.05 for all comparisons).

**Figure 4:**
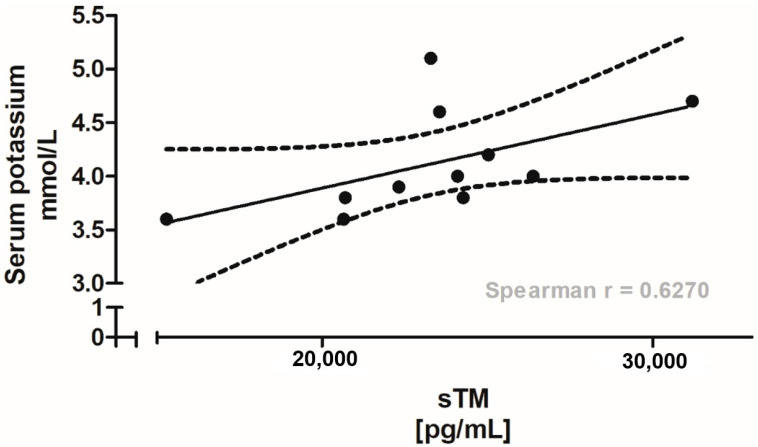
Relationship between plasma soluble thrombomodulin concentrations at 30 min post-reperfusion and serum potassium levels on day 7 post-transplantation in kidney transplant recipients from the HMP group (*n* = 20). The solid line represents the linear regression fit, with the 95% confidence interval (dotted lines). Individual points are shown. A significant positive correlation was observed (Spearman’s r = 0.6270; *p* = 0.034). Statistical analysis performed using Spearman’s rank correlation test (GraphPad Prism ver. 5).

In the HMP group, correlations between plasma sTM concentrations at 1 and 30 min post-reperfusion and post-transplant renal function parameters were assessed using Spearman’s rank correlation analysis. A significant positive correlation was identified between sTM concentrations at 30 min post-reperfusion and serum potassium levels on day 7 post-transplantation (*p* < 0.05). Linear regression analysis, with a 95% confidence interval, supported this association, suggesting that higher sTM levels may reflect endothelial injury and be associated with less favorable early graft function (Figure 4).

## 4. Discussion

Kidney transplantation remains the gold standard treatment for end-stage renal disease [18]. However, the increasing use of expanded criteria donors (ECDs) and donation after circulatory death (DCD) has increased the susceptibility of transplanted kidneys to ischemia–reperfusion injury, particularly at the endothelial level [18,19,20,21].

**Figure 5:**
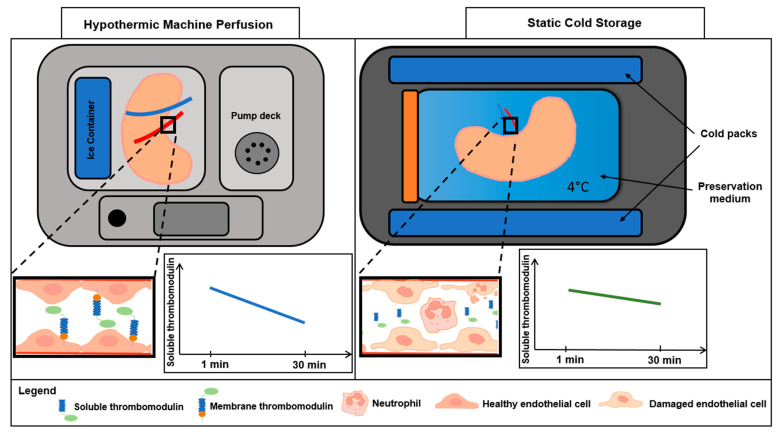
Conceptual overview of early endothelial responses in kidney preservation. Schematic representation of proposed differences in early graft-level endothelial responses associated with hypothermic machine perfusion and static cold storage during kidney transplantation. Hypothermic machine perfusion may influence microcirculatory perfusion and vascular resistance, thereby modulating ischemia–reperfusion-related endothelial injury (IRI). sTM, measured in the renal vein at 1 and 30 min post-reperfusion, is presented as a marker of early endothelial injury rather than as a predictor of clinical graft dysfunction.

IRI, an inevitable consequence of kidney transplantation, is a complex pathophysiological process that profoundly affects early graft function [22]. Endothelial injury plays a critical role in IRI by disrupting vascular homeostasis, promoting inflammation, and increasing thrombotic risk [23]. Soluble thrombomodulin, released from damaged endothelial cells, is a sensitive marker of endothelial injury and has been associated with post-transplant complications, including thrombosis and delayed graft function [18,24,25,26,27,28,29,30]. Figure 1 and Figure 5 schematically illustrate proposed differences in early endothelial responses associated with hypothermic machine perfusion and static cold storage, highlighting soluble thrombomodulin as a potential marker of ischemia–reperfusion-related endothelial injury during the very early post-transplant period.

In the present study, sTM concentrations decreased significantly between 1 and 30 min post-reperfusion in recipients of kidneys preserved by hypothermic machine perfusion, whereas no significant change was observed in kidneys preserved by static cold storage (Figure 2). The selection of the 1 and 30 min sampling time points was intended to capture the immediate post-reperfusion washout phase, during which endothelial-derived molecules are released from the graft microvasculature into the renal venous outflow, prior to systemic equilibration. Assessment at this stage allows evaluation of graft-level endothelial responses that are minimally influenced by recipient-related systemic factors.

The observed early decline in sTM levels in the HMP group suggests that HMP may attenuate endothelial injury during reperfusion, potentially through improved microcirculatory perfusion and reduced vascular resistance [31]. In contrast, stable sTM levels in the SCS group may reflect a greater degree of endothelial stress during the early post-transplant stage. The lack of statistically significant differences between groups at 30 min post-reperfusion may reflect the limited sample size. Although a correlation between sTM levels at 30 min and serum potassium on day 7 was observed, this finding should be interpreted with caution and considered exploratory. Notably, no significant differences in early renal function parameters were detected between groups, suggesting that the observed intra-group sTM dynamics reflect endothelial responses rather than short-term clinical superiority of one preservation strategy. Importantly, this early sTM dynamic was observed despite a significantly longer cold ischemia time in the HMP group, a factor generally associated with increased endothelial injury, suggesting that preservation-related effects may be underestimated rather than exaggerated in this cohort.

These observations are consistent with previous studies indicating that HMP influences early graft injury processes, although its long-term clinical advantages over SCS remain incompletely defined [32]. Meta-analyses, including that by O’Callaghan et al., have demonstrated that HMP reduces the incidence of DGF, particularly in kidneys from expanded criteria and donation after circulatory death donors; however, evidence for sustained long-term benefits remains inconclusive [33]. Interestingly, studies by Matos et al. and Gallinat et al. showed that HMP may mitigate DGF, even after prolonged SCS, highlighting its potential as a rescue strategy in marginal grafts [34,35]. In contrast, Kox et al. reported that the benefits of HMP are most pronounced when cold ischemia time is less than 10 h, with each additional hour increasing the risk of DGF by 8%, regardless of preservation method used [36]. These findings underscore that cold ischemia time remains a critical determinant of graft outcomes and suggest that the protective effects of HMP may be context-dependent rather than universal.

The endothelial-protective effects of HMP are likely mediated by improved microcirculatory perfusion and reduced vascular resistance. De Vries et al. demonstrated significant declines in vascular resistance within the first 30–60 min of perfusion, indicating rapid restoration of microvascular flow [31]. In addition to hemodynamic effects, HMP may attenuate inflammation by flushing pro-inflammatory cytokines (e.g., TNF-α, IL-6), as well as complement components from the graft microvasculature [37,38]. Together, these mechanisms may contribute to the distinct early sTM dynamics observed in the present study and support the concept that HMP modulates endothelial injury during the immediate reperfusion phase. However, the relative contribution of hemodynamic versus immunomodulatory effects remains unclear and warrants further investigation using integrated panels of endothelial and inflammatory biomarkers.

The potential therapeutic role of recombinant thrombomodulin (rTM) represents an important area for future research. Preclinical studies have demonstrated that rTM protects against acute kidney injury by improving microvascular perfusion and reducing thrombotic events [13]. In clinical settings, rTM has shown anti-inflammatory and anticoagulant effects in patients with sepsis-associated disseminated intravascular coagulation (DIC) [39]. In the context of kidney transplantation, rTM could theoretically mitigate IRI by protecting the endothelium and limiting microvascular thrombosis, thereby potentially reducing DGF and acute rejection. However, its application in human kidney transplantation remains unexplored, and further studies are required to establish its efficacy and safety. Although beyond the scope of the present study, therapeutic modulation of endothelial injury represents a promising translational research direction.

The present findings are built on limited prior research examining sTM in kidney transplantation. Ziętek et al. reported that elevated sTM concentrations in the renal vein after reperfusion were associated with post-transplant complications, including thrombosis, ureteral necrosis, and DGF, supporting the role of sTM as a marker of post-transplant risk [30]. Unlike measurements obtained from peripheral blood, renal vein sampling provides a direct assessment of graft-specific endothelial responses during the early post-reperfusion period. The present study extends these observations by demonstrating distinct very early sTM dynamics associated with different preservation strategies, highlighting the potential value of renal vein biomarkers for mechanistic studies of ischemia–reperfusion injury.

Despite its strengths, this study has several limitations. The relatively small sample size increases the risk of a type II error and may have masked biologically relevant between-group differences. Although the paired-kidney design substantially reduced donor-related biological variability, recipients differed between groups, and not all donor clinical variables were available in this retrospective cohort. In addition, the study was not designed or powered to assess the independent effects of cold ischemia time or donor type (expanded criteria versus standard criteria donors) on sTM levels, both of which may modulate the extent of endothelial injury. Future studies with larger cohorts, a longer follow-up, and more detailed donor profiling are essential to validate sTM as a biomarker and to elucidate the long-term benefits of HMP.

Emerging preservation strategies, such as normothermic machine perfusion (NMP), represent promising alternatives to HMP. By maintaining near-physiological conditions with oxygenated perfusates, NMP may further reduce IRI and enable ex vivo graft repair [40,41]. Experimental studies have demonstrated the feasibility of delivering pharmacological agents, gene therapies, and mesenchymal stromal cells during NMP, highlighting potential as a platform for graft optimization [42]. Integrating biomarkers, like sTM, into NMP protocols could facilitate real-time assessment of graft endothelial status and guide individualized preservation or therapeutic interventions.

## 5. Conclusions

In conclusion, this paired-kidney exploratory study provides insight into very early, graft-level endothelial responses during the reperfusion phase of kidney transplantation in relation to preservation strategies. Differences in early renal vein sTM dynamics suggest that the method of preservation may modulate endothelial behavior immediately after reperfusion. Although these findings do not establish clinical superiority of any preservation technique, they indicate that very early renal vein sTM measurements may be informative for studying ischemia–reperfusion-related endothelial injury. Given the limited sample size and exploratory nature of the study, further studies with larger cohorts, additional biomarkers, and extended follow-ups are required to determine the clinical relevance of these observations.

## Figures and Tables

**Table 1:** Clinical data of recipients before transplantation, qualified for the SCS and HMP groups.

No.	Clinical Data	SCS (*n* = 20)	HMP (*n* = 20)	Statistical Significance (*p*-Value)
1.	Sex: - Female - Male	10 (50%) 10 (50%)	7 (35%) 13 (65%)	0.23
2.	Age	Median 56.5 Min.–Max. 33–70	Median 54 Min.–Max. 34–68	0.35
3.	BMI	Median 27.8 Mean 27.9 Min.–Max. 19.4–36.8	Median 27.9 Mean 26.7 Min.–Max. 21.1–33.8	0.93
4.	Duration of hemodialysis (months)	Median 52 Mean 54 Min.–Max. 17–107	Median 47 Mean 40 Min.–Max. 3–83	0.32
5.	HLA mismatch A	Median 1.0 Mean 0.9 Min.–Max. 0–20	Median 1.0 Mean 1.2 Min.–Max. 0–2.0	0.43
6.	HLA mismatch B	Median 1.5 Mean 1.5 Min.–Max. 1.0–2.0	Median 1.0 Mean 1.2 Min.–Max. 0–2.0	0.32
7.	HLA mismatch DR	Median 1.0 Mean 0.9 Min.–Max. 0–1.0	Median 0.5 Mean 0.6 Min.–Max. 0–2.0	0.21
8.	Total HLA mismatch count	Median 3.0 Mean 3.25 Min.–Max. 2.0–5.0	Median 3.0 Mean 2.9 Min.–Max. 0–5.0	0.69
9.	HLA score (A2B5DR10)	Median 15.5 Mean 16.0 Min.–Max. 10.0–25.0	Median 20.0 Mean 20.0 Min.–Max. 5.0–34.0	0.15
10.	Serum creatinine (mg/dL)	Median 7.8 Mean 7.6 Min.–Max. 3.2–12.0	Median 7.2 Mean 7.6 Min.–Max. 3.4–12.2	0.89
11.	Serum urea (mg/dL)	Median 85.0 Mean 78.9 Min.–Max. 15.0–159.0	Median 97.0 Mean 98.2 Min.–Max. 27.0–152.0	0.17
12.	e-GFR (ml/min/1.73 m^2^)	Median 6.0 Mean 7.1 Min.–Max. 3.0–16.0	Median 7.0 Mean 7.7 Min.–Max. 4.0–13.0	0.46
13.	Serum potassium (mmol/L)	Median 4.6 Mean 4.6 Min.–Max. 4.3–4.8	Median 4.5 Mean 4.5 Min.–Max. 4.1–5.3	0.37
14.	WBC in blood (×10^3^/µL)	Median 6.5 Mean 8.3 Min.–Max. 5.4–19.5	Median 7.9 Mean 7.4 Min.–Max. 4.2–11.9	0.97
15.	Platelets (×10^3^/µL)	Median 191.0 Mean 245.3 Min.–Max. 139.0–686.0	Median 219.0 Mean 214.2 Min.–Max. 128.0–292.0	0.42
16.	Hematocrit	Median 0.35 Mean 0.35 Min.–Max. 0.3–0.4	Median 0.37 Mean 0.38 Min.–Max. 0.29–0.45	0.6

Abbreviations: BMI—body mass index; HLA—Human Leukocyte Antigen; e-GFR—estimated glomerular filtration rate; WBC—white blood cell.

## Data Availability

Data are available upon request.

## Supplementary Materials

The following supporting information can be downloaded at https://www.mdpi.com/article/10.3390/jcm15010269/s1. Table S1: Donor characteristics. Figure S1: Mean cold ischemia time of kidneys preserved using hypothermic machine perfusion (HMP) or static cold storage (SCS). Figure S2: Relationship between plasma soluble thrombomodulin concentrations at 30 min post-reperfusion and cold ischemia time (CIT) in (A) hypothermic machine perfusion (HMP) and (B) static cold storage (SCS) preserved kidneys (*n* = 20).

## Abbreviations

The following abbreviations are used in this manuscript:

BMI	Body mass index
CKD	Chronic kidney disease
CPP	Continuous pulsatile perfusion
DCD	Donation after circulatory death
DGF	Delayed graft function
ECDs	Expanded criteria donors
ESRD	End-stage renal disease
GFR	Glomerular filtration rate
HLA	Human leukocyte antigen
HMP	Hypothermic machine perfusion
IRI	Ischemia–reperfusion injury
mTM	Membrane-bound thrombomodulin
OPTN	Organ Procurement and Transplantation Network
PNF	Primary non-function
RRT	Renal replacement therapy
SDS	Static cold storage
sTM	Soluble thrombomodulin
UNOS	United Network for Organ Sharing
WBCs	White blood cells
