# Supplementary material for: Soluble Thrombomodulin as a Marker of Endothelial Injury in Early Post-Transplant Period: A Comparative Study of Simple Hypothermia and Pulsatile Machine Perfusion in Kidney Graft Preservation
Source: J Clin Med. 2025 Dec 29;15(1):269. doi: 10.3390/jcm15010269 (PMC12787036; doi:10.3390/jcm15010269)
Supplement: Supplementary file 1 [file jcm-15-00269-s001.zip › jcm-4012628-supplementary.pdf]

Soluble Thrombomodulin as a Marker of Endothelial Injury in Early Post-Transplant Period: A Comparative Study of Simple Hypothermia and Pulsatile Machine Perfusion in Kidney Graft Preservation

Maciej Kotowski [1,†], Anna Prekwa [1,†], Adam Nowacki [2], Iga Stukan [3,*], Karol Tejchman [1], Jerzy Sieńko [4], Przemysław Nowacki [5], Bogusław Machaliński [3] and Marek Ostrowski [1]

1 Department of General Surgery and Transplantation, Pomeranian Medical University, 70-111 Szczecin, Poland; maciej.j.kotowski@gmail.com (M.K.); annaprekwa@gmail.com (A.P.); ktejchman78@gmail.com (K.T.); mostrowski@poczta.onet.pl (M.O.)
2 Department of Vascular Surgery, General Surgery and Angiology, Pomeranian Medical University, 70-111 Szczecin, Poland; itskorn@gmail.com
3 Department of General Pathology, Pomeranian Medical University, 70-111 Szczecin, Poland; boguslaw.machalinski@pum.edu.pl
4 Department of General, Transplant and Liver Surgery, Pomeranian Medical University, 71-455 Szczecin, Poland; jsien@poczta.onet.pl
5 Department of Neurology, Pomeranian Medical University, 71, 252 Szczecin, Poland; nowackiprz@gmail.com
* Correspondence: iga.stukan@pum.edu.pl; Tel.: +48-91-466-1535
† These authors contributed equally to this work.

SUPPLEMENTARY

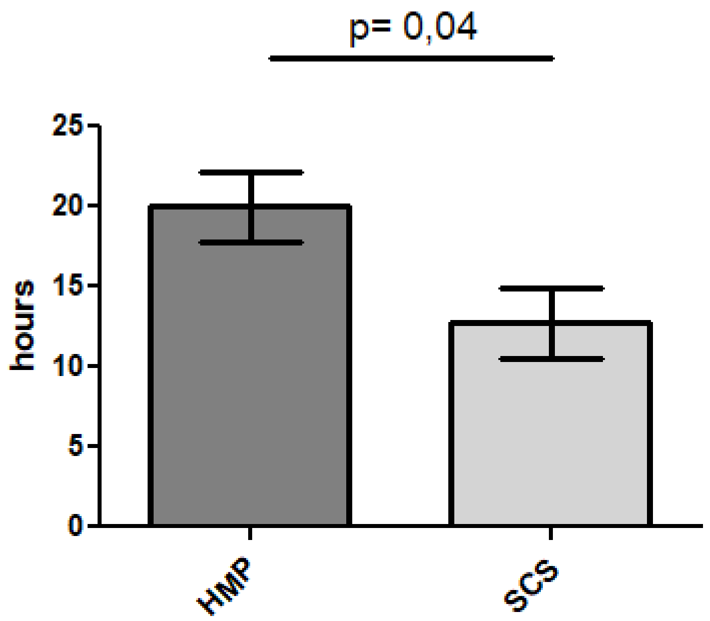

Figure S1. Mean cold ischemia time of kidneys preserved using hypothermic machine perfusion (HMP) or static cold storage (SCS). The results shown as mean ± SEM. T test was performed using GraphPad Prism version 5.00 for Windows, GraphPad Software, San Diego California USA, www.graphpad.com

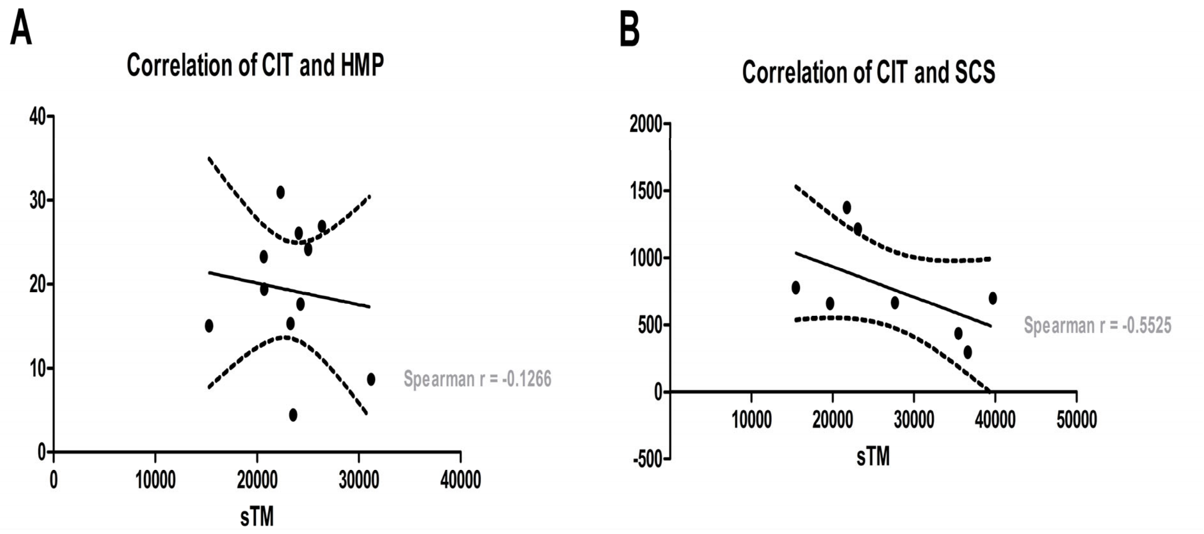

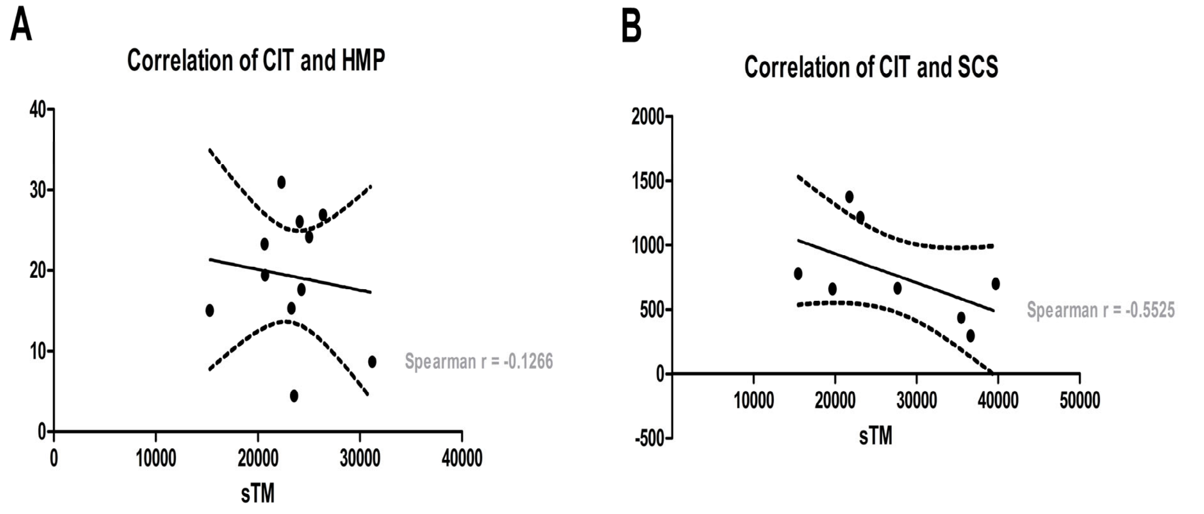

Figure S2. Relationship between plasma soluble thrombomodulin concentrations at 30 minutes post-reperfusion and cold ischemia time (CIT) in (A) hypothermic machine perfusion (HMP) and (B) static cold storage (SCS) preserved kidneys (*n*=20). The solid line represents the linear regression fit, with the 95% confidence interval (dotted lines). Individual points are shown. Spearman's rank correlation test was performed using GraphPad Prism version 5.00 for Windows, GraphPad Software, San Diego California USA, www.graphpad.com

Table S1. Donor characteristics.

Variable	Donors (n = 22)
**Age, years**	50.6 ± 11.7
**Male sex, *n* (%)**	10 (45%)
**ABO blood group, n (%)**	
**– O**	8 (36%)
**– A**	6 (27%)
**– B**	5 (23%)
**– AB**	5 (23%)
